# Microstructure and Chemical Stability of Al_2_O_3_-ZrO_2_-ReB_2_ Composite Coatings Obtained by Air Plasma Spraying

**DOI:** 10.3390/ma18143363

**Published:** 2025-07-17

**Authors:** Adriana Wrona, Kinga Czechowska, Katarzyna Bilewska, Monika Czerny, Anna Czech, Marcin Lis, Anna Brudny, Grzegorz Muzia, Lucyna Jaworska

**Affiliations:** 1 Łukasiewicz Research Network—Institute of Non-Ferrous Metals, Sowinskiego 5 St., 44-100 Gliwice, Poland; adriana.wrona@imn.lukasiewicz.gov.pl (A.W.); kinga.czechowska@imn.lukasiewicz.gov.pl (K.C.); katarzyna.bilewska@imn.lukasiewicz.gov.pl (K.B.); monika.czerny@imn.lukasiewicz.gov.pl (M.C.); anna.czech@imn.lukasiewicz.gov.pl (A.C.); marcin.lis@imn.lukasiewicz.gov.pl (M.L.); anna.brudny@imn.lukasiewicz.gov.pl (A.B.); grzegorz.muzia@imn.lukasiewicz.gov.pl (G.M.); 2 Department of Physical Metallurgy and Powder Metallurgy, Faculty of Metals Engineering and Industrial Computer Science, AGH University of Krakow, Mickiewicza 30 Av., 30-059 Krakow, Poland

**Keywords:** atmospheric plasma spray, Al_2_O_3_-ZrO_2_-ReB_2_, coatings, agglomeration, XRD, microstructures, chemical stability

## Abstract

This study investigated the effect of adding superhard ReB_2_ to atmospheric plasma sprayed (APS) coatings based on 60 wt% Al_2_O_3_ and 40 wt% ZrO_2_. The amorphous phases commonly present in such coatings are known to impair their performance. ReB_2_ was introduced as a crystallization nucleus due to its high melting point. ReB_2_ decomposes in the presence of moisture and oxygen into H_3_BO_3_, ReO_3_, HBO_2_, and HReO_4_. ReB_2_ was encapsulated with Al_2_O_3_ via metallothermic synthesis to improve moisture stability, yielding a powder with d_90_ = 15.1 μm. After milling, it was added at 20 wt% to the Al_2_O_3_-ZrO_2_ feedstock. Agglomeration parameters were optimized, and coatings were deposited under varying APS conditions onto 316L steel substrates with a NiAl bond coat. In the coating with the highest ReB_2_ content, the identified phases included ReB_2_ (2.6 wt%), Re (0.8 wt%), α-Al_2_O_3_ (30.9 wt%), η-Al_2_O_3_ (32.4 wt%), and monoclinic and tetragonal ZrO_2_. The nanohardness of the coating, measured using a Vickers indenter at 96 mN and calculated via the Oliver–Pharr method, was 9.2 ± 1.0 GPa. High abrasion resistance was obtained for the coating with a higher content of η-Al_2_O_3_ (48.7 wt%). The coefficient of friction, determined using a ball-on-disc test with a corundum ball, was 0.798 ± 0.03. After 15 months, the formation of (H_3_O)(ReO_4_) was observed, suggesting initial moisture-induced changes. The results confirm that Al_2_O_3_-encapsulated ReB_2_ can enhance phase stability and crystallinity in APS coatings.

## 1. Introduction

The development of new coating materials with high hardness and enhanced abrasion resistance, applicable via industry-standard methods such as Atmospheric Plasma Spraying (APS), remains a critical area of research [1]. APS coatings, particularly those based on Al_2_O_3_–ZrO_2_ mixtures, are commonly used for components exposed to abrasive, erosive, and corrosive conditions. However, these coatings often exhibit conventional lamellar microstructures with inherent defects, including pores, cracks, and partially or unmelted particles [2,3,4,5,6]. Moreover, APS processing of Al_2_O_3_–ZrO_2_ frequently results in the formation of an amorphous phase, which has been shown to reduce coating hardness [7,8,9,10,11].

The aim of the following studies was to introduce particles of ReB2 that will promote the crystallization of Al_2_O_3_–ZrO_2_ coating with a near-eutectic composition [12]. ReB_2_ is a superhard compound with a high melting point. ReB_2_ (P6_3_/mmc), originally synthesized in 1962 [13], has demonstrated hardness values exceeding 40 GPa [14,15]. Its high resistance to elastic and plastic deformation arises from strong covalent B–B and Re–B bonds [16]. Among several polymorphic forms, the hexagonal P6_3_/mmc structure is considered the most thermodynamically stable [16,17].

Despite its mechanical advantages, ReB_2_ is known to react with moisture, leading to degradation products such as H_3_BO_3_ and HBO_4_. These acids are hygroscopic and form a liquid layer on the surface of the ReB_2_ material, which further contributes to the destruction of the bulk material [18,19]. Recent studies have shown that passivation with aluminum or titanium, either through doping or encapsulation, can significantly enhance its chemical stability [20,21]. For instance, thin films of Re–Al alloys have demonstrated improved resistance to atmospheric degradation due to the formation of protective Al_2_O_3_ layers [20].

ReB_2_-based coatings have been produced using methods including Confined-Plume Chemical Deposition, magnetron sputtering, and pulsed laser deposition [20,21,22,23,24,25]. However, their integration into plasma-sprayed ceramic systems remains underexplored. The decreasing cost of rhenium and advances in recycling technologies further support the feasibility of incorporating ReB_2_ into industrial coating processes [26].

The present research addresses this gap by investigating the effect of ReB_2_ incorporation on the structure and properties of APS-deposited Al_2_O_3_-ZrO_2_ coatings. The study involved the preparation of composite feedstock powders, optimization of the spraying process, and characterization of the resulting microstructures and phase compositions. The primary objective was to develop a superhard, moisture-resistant Al_2_O_3_-ZrO_2_-ReB_2_ coating material with minimized amorphous content, suitable for use in wear-resistant ceramic coatings produced via thermal spraying.

## 2. Materials and Methods

### 2.1. Initial Materials—ReB_2_ + Al_2_O_3_ Powders Obtaining

In order to obtain a composite material consisting of rhenium boride and alumina, the process of metallothermic reduction of rhenium oxide using Al was used [27]. The starting materials for obtaining the ReB_2_ + Al_2_O_3_ composite powder at elevated temperature in an Ar gas shield were: ammonium perrhenate (APR)—NH_4_ReO_4_ (99.9%, INNOVATOR SP.z o.o., Gliwice, Poland) as a substrate to obtain ReO_2_ and Re precursor; aluminum powder (99.8%, Al BLS0072:APS7/99.8 AlATOMIZED:CJ01, Benda-Lutz Skawina Sp. z o.o., Skawina, Poland) as a stoichiometric reductant being consumed during the process for the metallothermic reaction; B_2_O_3_ powder (~420 μm, 98%, AcrosOrganics, Jinan, China) as a boron precursor; and Al_2_O_3_ powder (≥99.8%, Areoxide Alu C, BET: 85–115 m^2^/g, Evonic Industries AG, Essen, Germany) to slow down the reaction [28]. It was assumed that the metallothermic reaction would take place according to reaction (1):

3 MeO_2_ + 3 B_2_O_3_ + 10 Al → 3 MeB_2_ + 5 Al_2_O_3_ (1)

Me can be substituted with a specific metal, e.g., Re.

The synthesis of these materials is described in detail in [28]. ReO_2_, as the substrate used in reaction (1), was obtained by the thermal method from ammonium perrhenate, through two-stage thermal treatment, in a tube electric furnace, at a temperature of 350 °C, for 1.5 h, in an argon flow of 1 L/min (first stage) and 470 °C, for 1.5 h, in an argon flow of 1 L/min (second stage). Meanwhile, ReB_2_ from reaction (1) was obtained at a temperature of 1050 °C, for 1 h, in an argon flow of 4 L/min. Materials with a higher content of Al_2_O_3_ and Al produced in syntheses at a molar ratio B_2_O_3_/ReO_2_ = 1 are characterized by greater chemical stability and lower reactivity with water than materials with lower Al_2_O_3_ and Al content [28]. Quantitative and qualitative analysis of the phase composition of the composite powder was carried out. For X-ray diffraction measurements, an XRD7 Seifert-FPM diffractometer (Freiberger Präzisionsmechanik, FPM Holding GmbH, Freiberg, Germany) was employed using CuKα*_1_* radiation with a wavelength of λ = 1.540598 Å and the Ni filter. Measurements were performed in the Bragg–Brentano geometry. The scan was performed with a step size of 0.04°. Phase identification was carried out using software Match! V.4.1. in conjunction with the ICDD PDF -5+ database (2025 update). The following reference cards were applied for phase analysis: ReB_2_—04-008-5058, 01-078-4108; Al_2_O_3_—04-006-9359; Re_7_B_3_—04-004-2746. A semi-quantitative evaluation of the phase composition was conducted in Match! 3 software using the Reference Intensity Ratio (RIR) method, comparing the intensity scaling factors of identified phases against a corundum standard.

**Figure 1:**
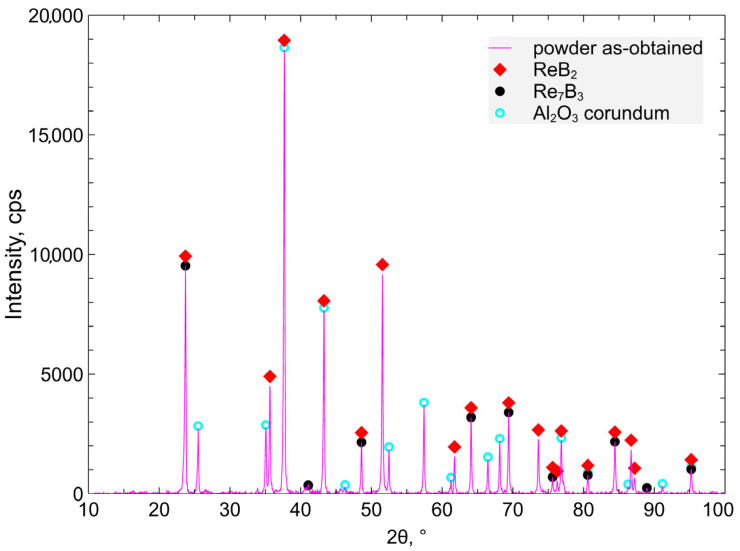
The XRD diffractogram of the synthesized ReB_2_ + Al_2_O_3_ powders.

The XRD phase composition of the ReB_2_ + Al_2_O_3_ powder measurements is presented in Figure 1.

**Figure 2:**
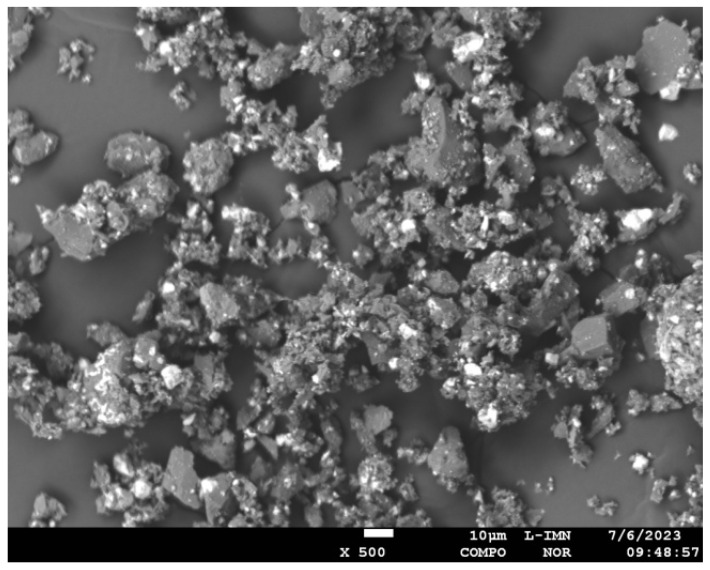
Morphology of the composite ReB_2_ + Al_2_O_3_ powders, COMPO image, magnification 500×.

Synthesized powders were analyzed by Scanning Electron Microscopy (SEM) using JEOL JXA 8230 (X-Ray microprobe, JEOL Ltd., Tokyo, Japan). Figure 2 illustrates the morphology of composite powders Al_2_O_3_ + ReB_2_ after the crushing and milling processes.

The milling was carried out in a planetary ball mill type PM400 by Retsch (Fisher Scientific, Waltham, MA, USA) in bowls (250 mL) with a ZrO_2_ lining, using 10 mm diameter ZrO_2_ balls. The powder fraction after the crushing process (<312 µm), with the addition of ethyl alcohol, was introduced into the mill bowl. The milling was carried out for 40 min, with a milling speed of 180 rpm. The ratio of the powder mass (subjected to a single milling operation during 40 min.) to the mass of the balls was 1:3. Densities were measured using an AccuPyC II 1340 gas pycnometer (Micromeritics, part of the Malvern Panalytical Ltd., Westborough, MA, USA). The specific surface area of the powders was measured by the BET method using Gemini 2360 (Micromeritics Instrument Corporation, Norcross, GA, USA) surface area analyzer, (Table 1).

The powder was tested after 20 months of storage in a container (polypropylene) filled with argon. Boric acid H_3_BO_3_ and aluminum rhenium oxide hydrate Al(ReO_4_)_3_(H_2_O)_8_, along with the phases ReB_2_, corundum Al_2_O_3_, and Re_7_B_3_, were observed in the XRD analysis of this powder.

### 2.2. Agglomeration of Composite Powders

Studies were carried out on agglomerates using the so-called ceramic binding phases based on 80 wt% of Al_2_O_3_-ZrO_2_ powders (60:40, Amperit 750, fused, grain size 22/5 µm, d_10_ = 5.66 µm, d_50_ = 15.93 µm, d_90_ = 35.35 µm, prod. H.C. Strack, now Höganäs AB, Höganäs, Sweden), 20 wt% of the synthesized ReB_2_ + Al_2_O_3_ powder with the addition of a binder in the form of an aqueous solution—an aqueous solution of 10, 15, or 20 wt% hydroxypropylcellulose (HPC-SL Fine Powder, Nippon Soda Co., Ltd., Tokyo, Japan) with a concentration of 5%. The synthesized Al_2_O_3_ + ReB_2_ powder was characterized by a particle size similar to that of the commercial Al_2_O_3_-ZrO_2_ powder. Size enlargement by wet agglomeration is widely used in the powder processing industry. In this work, the wet shear agglomeration method was used, employing a high shear mixer type Eirich EL1 (Maschinenfabrik Gustaw Eirich GmbH & Co KG, Hardheim, Germany). The process consists of mixing, densifying, and agglomerating wet powder particles under the action of shearing and densifying forces exerted by the impeller in high-speed mixers [29]. Agglomeration is necessary for technological reasons of the APS process and helps to distribute the elements evenly in the coating. The criterion for selecting the amount of binder added was to create as many larger particles as possible in the agglomerates.

**Figure 3:**
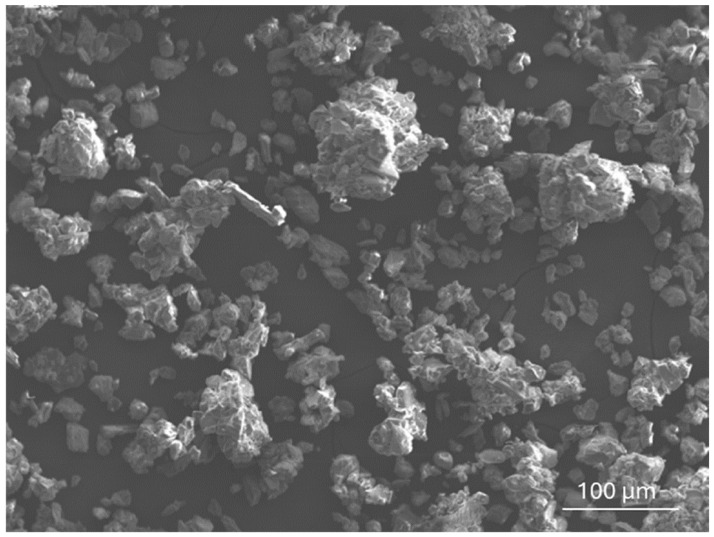
Morphology of the composite 80 wt% Al_2_O_3_-ZrO_2_ and 20 wt% ReB_2_ + Al_2_O_3_ powder < 100 µm (SEI, magnification 200×).

**Figure 4:**
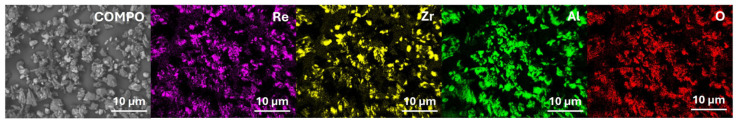
Element distributions in agglomerates, obtained from 80 wt% Al_2_O_3_-ZrO_2_ + 20 wt% Al_2_O_3_ + ReB_2_ powder.

Figure 3 shows an example of agglomerates produced using a high-speed mixer (fraction immediately after agglomeration) and after the grinding and sieving process. In Figure 4, the elements distribution is shown.

Table 2 presents results of agglomerate compositions for 80 wt% Al_2_O_3_-ZrO_2_, 20 wt% Al_2_O_3_ + ReB_2_ with 10 wt% of binder in the form of an aqueous solution of hydroxypropylcellulose.

## 3. Results of the Atmospheric Plasma Spraying Process

The coating spraying process was carried out at the laboratory stand, using the AP 50 plasma spray system with an F4 plasma spray gun torch, equipped with the PF-50 powder feeder (FST-Flame Spray Technologies, Duiven, The Netherlands). The coatings were sprayed onto sheets made of 316L stainless steel with dimensions of 190 mm × 85 mm × 5 mm. The sheets were previously cut using the water-jet method to prepare smaller shapes in the sheet—samples in the form of squares with dimensions of 35 mm × 35 mm (which were cut out of the sheet after spraying the coating). Before spraying the coatings, the metal sheets were subjected to sandblasting to increase the adhesion of the substrate surface and spraying an intermediate coating, which was NiAl (Amperit 281—NiAl 95-5, gas atomized, 90 + 45 µm, H.C. Starck, now Höganäs AB, Höganäs, Sweden) powder material. Parameters for the process are presented in Table 3.

APS processes for the 80 wt% Al_2_O_3_-ZrO_2_, 20 wt% Al_2_O_3_ + ReB_2_ coatings are realized using the following constant values: current intensity—600 A; distance of the torch nozzle from the steel substrate (on which the coatings were applied)—120 mm; number of powder spray repetitions on the substrate (i.e., torch passes)—four times; and powder feeder—5.5 rpm. The share of H_2_ and Ar during the atmospheric plasma spraying process is presented in Table 4.

The Ar/H_2_ ratio affects, among other things, the plasma temperature and the shape of the plasma jet. In coating samples no. 2 and no. 3 (Table 4 and Table 5), which were sprayed using a higher flow of hydrogen as a process gas, losses in the form of splatters were observed on the coating surface. In these samples, the lowest content of the ReB_2_ phase and the α-Al_2_O_3_ phase, and the ZrO_2_—monoclinic phase were noted based on the XRD test results. These samples, however, were characterized by the highest content of the η-Al_2_O_3_ phase (over 60% by weight), which is a cubic form of alumina characterized by a large surface area, high hardness, and good thermal stability [30]. It is a metastable phase, meaning it can transform into the more stable alpha-alumina (α-alumina) under certain conditions. Sample no. 4 is characterized by a high content of the η-Al_2_O_3_ phase (about 48.7 wt%) and of ReB_2_ and Re, slightly lower than samples no. 5 and 6 (Table 5). A higher hydrogen content, H_2_, may affect ReB_2_ decomposition processes. The selection of the Ar/H_2_ ratio was based on obtaining a coating with the highest content of the ReB_2_ phase. Based on the results of quantitative XRD phase composition analysis, it was found that it is advantageous to use a larger flow of argon as the process gas relative to the flow of hydrogen.

### 3.1. Microstructure of APS Coatings

SEM surface microstructural studies of the coatings were realized using a Zeiss LEO Gemini 1525 electron microscope (Carl Zeiss Microscopy GmbH, Jena, Germany) with a Bruker Quantax XFlash^®^ 5010 EDS SDD detector (Bruker, Rheinstetten, Germany) and an X-ray microprobe JXA 8230 JEOL (JEOL Ltd., Tokyo, Japan). SEM microstructural studies of cross-section coatings were realized using a Zeiss Evo MA10 electron microscope (Carl Zeiss Microscopy GmbH, Jena, Germany) with a Bruker Quantax XFlash^®^ 6 Bruker Nano (EDS SD) detector (Bruker, Rheinstetten, Germany) and a JXA 8230 JEOL X-ray microprobe (JEOL Ltd., Tokyo, Japan).

**Figure 5:**
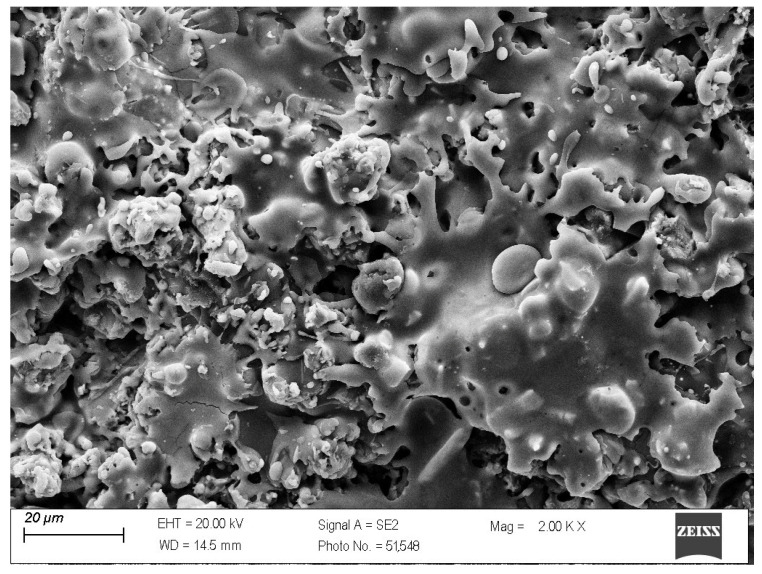
The surface of the 80 wt% Al_2_O_3_-ZrO_2_ with 20 wt% Al_2_O_3_ + ReB_2_ APS coating (magnification 2000×).

The microstructure of the surface of coating no. 5 (Table 5) with the highest content of ReB_2_ and α-Al_2_O_3_ is presented in Figure 5.

**Figure 6:**
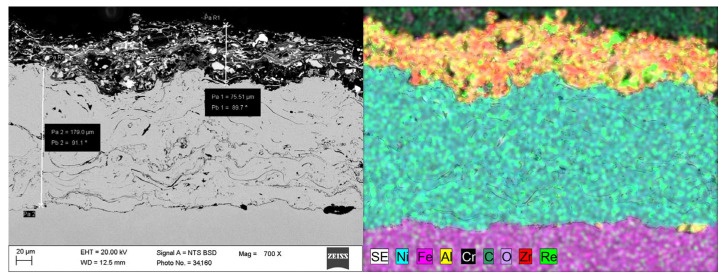
The elements distribution (EDS) for the cross section of the 80 wt% Al_2_O_3_-ZrO_2_ with 20 wt% Al_2_O_3_ + ReB_2_ APS coating, sample number 5 (Table 5), magnification 700×.

**Figure 7:**
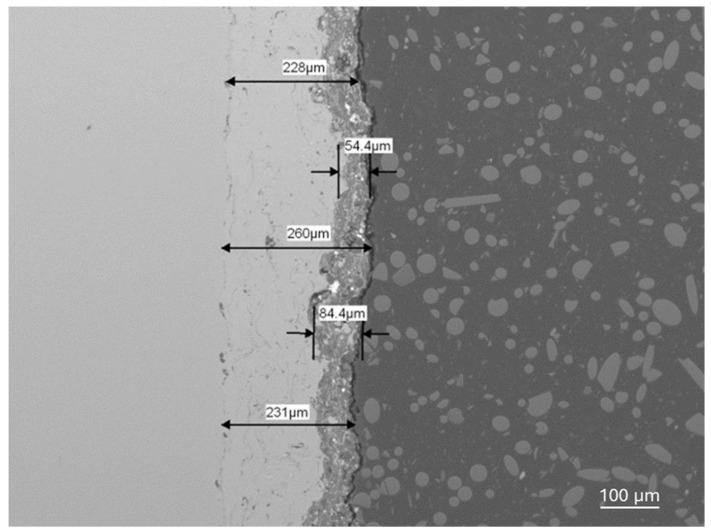
The Al_2_O_3_ + ZrO_2_ + ReB_2_ + Re coating and the NiAl subcoating thicknesses for sample number 5 (Table 5), magnification 100×.

SEM and EDS microstructural examination of the cross-section of coating number 5 is presented in Figure 6 and Figure 7.

The thickness of coating no. 4 is about 100 μm. Coatings no. 5 and no. 6 (Table 5) have very similar phase compositions but differ in thickness. Coating no. 5 is approximately 80 μm thick, while coating no 6 (Table 5) is approximately 35 μm thick. The nanohardness tests were carried out using a KLA nanoindenter model G200 (Keysight Technologies, Santa Rosa, CA, USA) Hardness was calculated using the Oliver–Pharr method. Measurements were taken with a Vickers indenter with a maximum force of 96 mN. The nanohardness of coatings no. 4, no. 5, and no. 6 are, respectively, 937 ± 15, 919 ± 10, and 451 ± 10 (Table 5). The phase composition of samples No. 5 and No. 6 is very similar, but the coatings differ in thickness by more than two times. The relationship between hardness and coating thickness is clearly visible in this case. The HV0.1 nanohardnesses are lower than for the Al_2_O_3_ + ZrO_2_ coatings, which are about 1070, but the thickness of these the Al_2_O_3_ + ZrO_2_ coatings is about three times higher, which influences the stiffness of the system and the measurement result [11].

### 3.2. Ball-on-Disk Studies of the APS Coating

**Figure 8:**
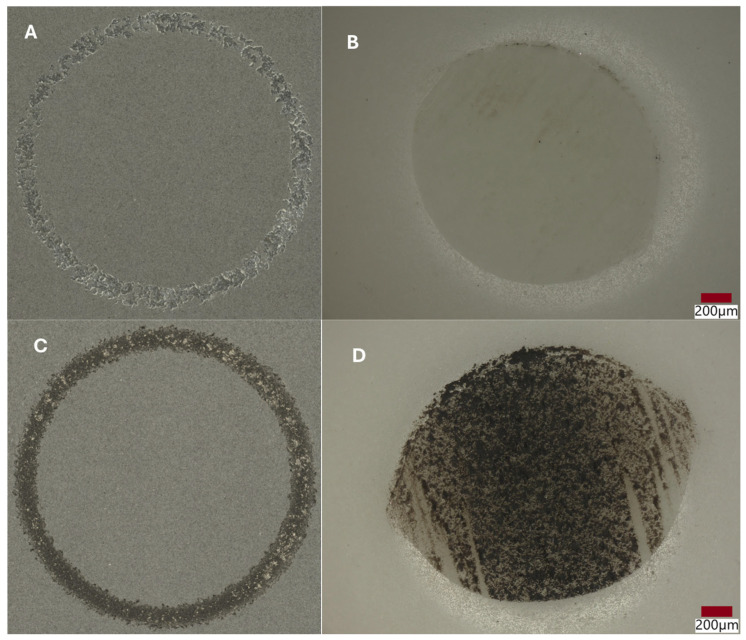
Wear tracks of abrasions on the coatings and counter samples (balls), examined using an optical microscope after tribological tests: (**A**,**B**) coating no. 4; (**C**,**D**) coating no. 5.

Ball-on-disk tribological tests (tribotester, THT CSM Instruments, part of the Anton Paar Group, Graz, Austria) were realized for the Al_2_O_3_ + ZrO_2_ + ReB_2_ + Re coatings (for samples no. 4 and no. 5). For comparison, studies were carried out for 316L (no. 1.4404, EN 10028-7 [31]) steel. Counter samples were Al_2_O_3_ balls, and the diameter of the balls was Ø6 mm. Sampling frequency during tests was 60 Hz, rub radius—6 mm, load 15 *n*, linear speed—10 cm/s, rotation speed—95.4 rpm, friction path—600 m. The roughnesses of the coating surfaces were measured using a Hommel-Etamic W20 profilometer (Jenoptic AG, Jena, Germany). The roughness coefficient Ra for sample no. 4 is 10.07 μm, and for sample no. 5, the Ra is 10.36 μm. Track analyses, along with the analysis of the wear profile of the samples after tribological tests, were performed on a Keyence VHX-7000 digital microscope (MC Keyence, Mechelen, Belgium). In Figure 8, the wear traces on coating surfaces and balls are presented.

**Figure 9:**
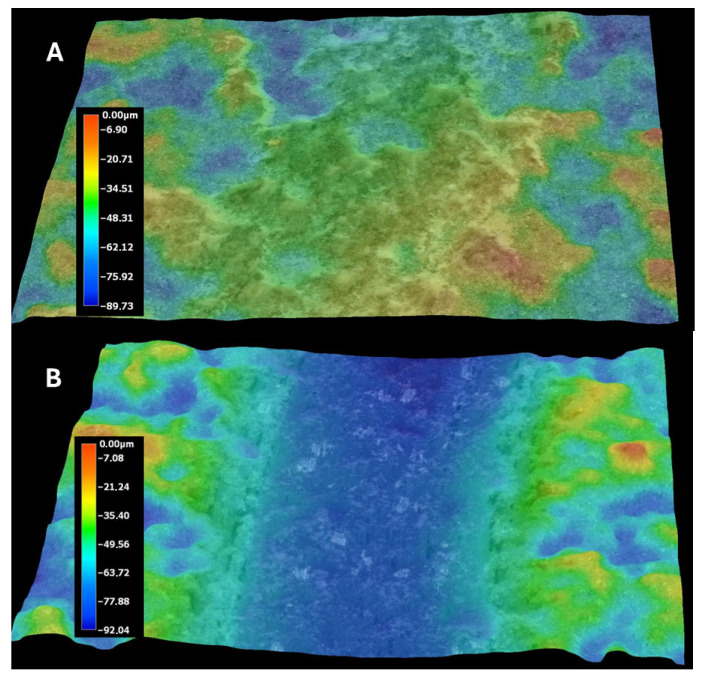
Wear tracks of abrasions on the coatings: (**A**) sample no. 4; (**B**) sample no. 5.

Figure 9A,B presents the profiles for the wear tracks of sample no. 5 after the ball-on-disk dry tests with the Al_2_O_3_ counterpart.

**Figure 10:**
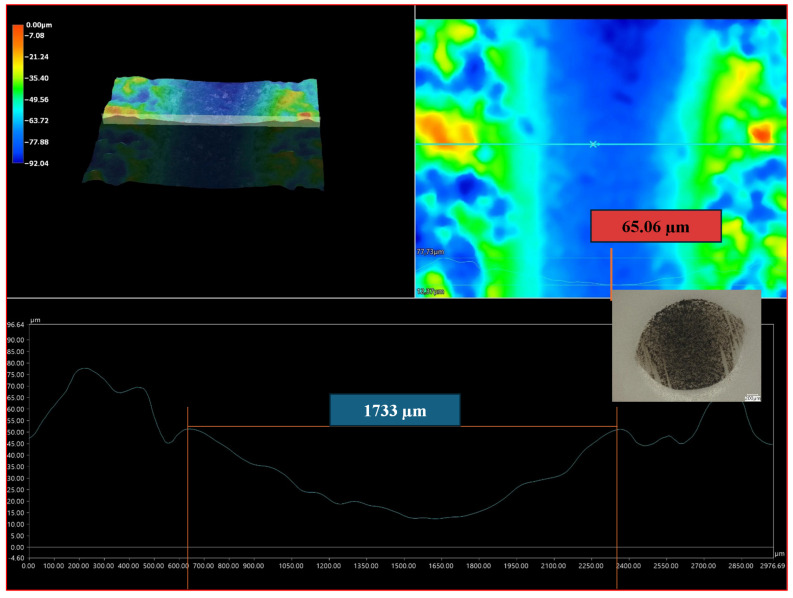
Profiles for the wear track of sample no. 5, after the ball-on-disk dry test with the Al_2_O_3_ counterpart. The profile shown at the bottom of the figure corresponds to the line on the upper right side of the figure.

Figure 10 presents the profiles for the wear tracks of sample no. 5 after the ball-on-disk dry tests with the Al_2_O_3_ counterpart.

**Figure 11:**
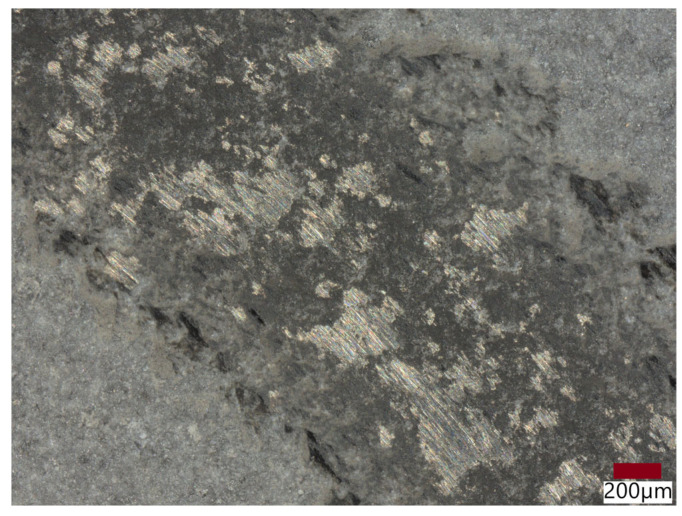
Microstructure of the wear scar of sample no. 5, after the ball-on-disk test.

The wear process is influenced by the abrasive mechanism, mainly the micro-cutting sub-mechanism. Figure 11 clearly shows the scratches in the wear scar of sample no. 5.

Average coefficients of friction and samples mass loss for coatings no. 4 and 5 and the 316L steel are presented in Table 6.

### 3.3. Chemical Stability of the Al_2_O_3_ + ZrO_2_ + ReB_2_ + Re Coating

**Figure 12:**
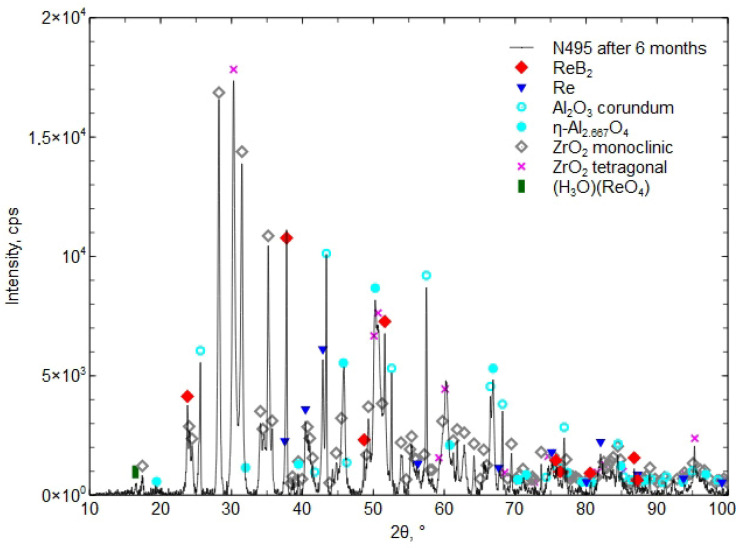
The XRD phase composition of the Al_2_O_3_ + ZrO_2_ + ReB_2_ + Re coating after six months of storage in a plastic bag.

In Figure 12, the XRD phase composition of the Al_2_O_3_ + ZrO_2_ + ReB_2_ + Re coating after six months of storage at atmospheric pressure, in a plastic bag (polietylen PE-LD), is presented.

**Figure 13:**
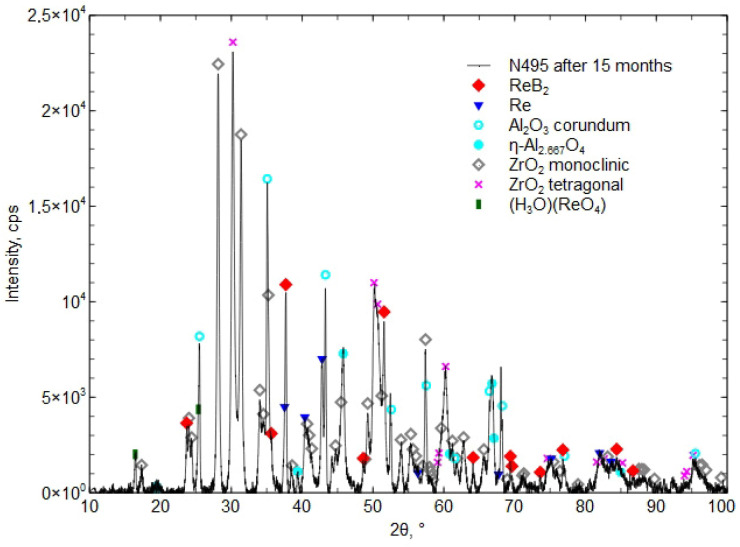
The XRD phase composition of the Al_2_O_3_ + ZrO_2_ + ReB_2_ + Re coating after 15 months of storage in a plastic bag.

Figure 13 presents the XRD phase composition of the Al_2_O_3_ + ZrO_2_ + ReB_2_ + Re coating after 15 months of storage.

After 6 and 15 months, the coating was found to contain the hydronium rhenium oxide (H_3_O)(ReO_4_) [04-012-4121], which indicates a reaction with water. The presence of the boric acid H_3_BO_3_ phase was not detected, which indicates its small amount, below the detection limit of the XRD method (less than 2%).

## 4. Discussion

Research carried out by other scientists has shown that for the Al_2_O_3_-ZrO_2_ composite coatings obtained using APS, an amorphous phase was formed due to the rapid cooling and solidification of plasma spraying [11]. If the coating has a higher amorphous phase content, it always undergoes a greater volume reduction factor when operating at high temperatures, which is directly related to crystallization [32]. The change in the volume of the coatings during their operation contributes to a reduction in their mechanical properties. The introduction of crystallization nuclei, particularly high-melting particles of other phases, can facilitate the transformation of amorphous phases into crystalline structures. These nuclei act as centers for crystal growth, leading to the transformation of the surrounding amorphous material into a crystalline structure [33]. The X-ray diffraction pattern presented in Table 5 confirms the crystalline structure of the obtained coatings. The addition of high-melting rhenium boride in the present work was aimed at improving the mechanical properties of the coatings and introducing crystal nuclei into the applied coating in order to obtain the crystal structure of the entire Al_2_O_3_-ZrO_2_ coating. Rhenium diboride is a good candidate for nucleation of the crystallization process and improvement of the properties of Al_2_O_3_-ZrO_2_ coatings due to its high melting point, high hardness, and fracture toughness. However, it has the disadvantage of being hygroscopic. Reports in the literature indicate that encapsulation with Al_2_O_3_ can reduce or eliminate the reaction of ReB_2_ with water [20].

For the synthesis of Al_2_O_3 +_ ReB_2_, the metallothermic reaction was used, with participation of aluminum as the stoichiometric reductant being consumed during the process. Past studies confirmed that the molar ratio B_2_O_3_/ReO_2_ = 1 guarantees the highest percentage of ReB_2_ in the product and better water resistance compared to syntheses with other B_2_O_3_/ReO_2_ ratios [28]. The composite material composition after synthesis is α Al_2_O_3_—75.8%, ReB_2_—23.2%, Re_7_B_3_—1% (Figure 1 and Figure 2, Table 1). After the material crushing and milling, the grain size d_90_ is 15.1 μm (90% of the particles have a diameter less than or equal to the indicated value).

Al_2_O_3_-ZrO_2_ is the initial matrix material of the APS coating, which constitutes 80 wt% of the coating material. The composition of this mixture was selected as 60 wt% Al_2_O_3_ and 40 wt% ZrO_2_. It is near the eutectic composition for the binary equilibrium phase diagram of the zirconia–alumina system [7]. According to previous studies, the flowability of the 60 wt% Al_2_O_3_ and 40 wt% ZrO_2_ melt was better than for other weight ratios for Al_2_O_3_ and ZrO_2_ APS composite coatings (35 wt% and 45 wt% of ZrO_2_) [11]. The addition of 20 wt% Al_2_O_3_—ReB_2_ composite powder was aimed at reducing the presence of the amorphous phase in the coating and increasing the abrasion resistance of the ReB_2_-containing coating.

Shear agglomeration was used to prepare agglomerates intended for the APS, which enables the transformation of fine, cohesive powders into strong, dense agglomerates. The quality of agglomerates depends on the quantity and viscosity of the binders and the grain size of the powders used [34]. Different amounts of binder—10 wt%, 15 wt%, and 20 wt%—were used in the form of hydroxypropylcellulose. The initial grain size of the commercial Al_2_O_3_-ZrO_2_ powders, d_90_, is 35.35 μm. The best agglomerate size distribution was obtained for 10 wt% of binder, which is shown in Table 2. Agglomerates are characterized by a growth characteristic of a layering mechanism, which is presented in Figure 3. The reason for the layered morphology of the agglomerates is the coarse powder. Despite this layered morphology, the agglomerates are characterized by a uniform distribution of elements, which is visible in Figure 4. In order to obtain better adhesion on steel substrates (316L), NiAl was deposited, and then the obtained agglomerates were deposited using different APS process parameters, as shown in Table 4. The choice of process parameters was determined by the largest share of ReB_2_ after the APS process. The results of the coating composition are presented in Table 5. The highest ReB_2_ content was characterized by samples 5 and 6. The thickness of the NiAl coating was about 230 μm (Figure 7). The share of ReB_2_ in coatings no. 5 and 6 is about 2.6 wt%. For these same materials, monoclinic and tetragonal ZrO_2_ were obtained. A commercial non-stabilized mixture was used for the tests; in future works, yttria-stabilized tetragonal ZrO_2_ (Y-TZP) should be used, which will be more beneficial for the phase stability of the coating. Y-TZP is outstanding in terms of low thermal conductivity and has good thermal shock resistance [35]. No amorphous phase was observed in the materials, which may have a positive effect on the abrasion resistance of new coatings (Table 6). The surface of the APS coating is very rough. Figure 5 confirms the presence of fully and partially melted powders. The elements distribution on the cross section confirms the presence of large rhenium or ReB_2_ particles (see Figure 6).

ReB_2_ has a very high melting point, about 2400 °C, and a very low self-diffusion coefficient [36]. The coatings are characterized by the porosity typical of APS coatings. Voids are visible in Figure 6. The larger amount of ReB_2_ particles accumulates at the bottom of the coating (green spots in Figure 6), although single unmelted particles are also visible on the surface. Pores occur in contact with the particles. The reason for the accumulation of pores near ReB_2_ may be the large unmelted ReB_2_ particles falling to the Al_2_O_3_-ZrO_2_ coating bottom (due to their high density).

Abrasion resistance tests were carried out. Because the microstructure of APS coatings is characterized by high porosity, a lamellar structure, and unmolten particles, the wear properties of APS coatings consequently depend strongly on spraying conditions (current intensity, distance of the torch nozzle from the substrate, share of gases, and gas flow) that affect the plasma temperature and on variables related to the injected powder (particle size, feeding rate), as well as the substrate’s mechanical properties [37]. The coatings were deposited under various conditions shown in Table 5. Coating no. 4 is characterized by the high ReB_2_ + Re content and a high η-Al_2_O_3_ content (1.2 wt%—ReB_2_, 1.5 wt%—Re, 14.2 wt%—α-Al_2_O_3_, 48.7 wt% η-Al_2_O_3_, 19.0 wt%—monoclinic ZrO_2_., 15.5 wt%—tetragonal—ZrO_2_). The η-Al_2_O_3_ phase has good mechanical properties [30], making coating no. 4 very abrasion resistant, as evidenced by the low mass loss in the ball-on-disk test, presented in Table 6 and the wear track abrasion profile presented in Figure 8A,B. The wear properties of coating no. 5 (2.6 wt%—ReB_2_, 0.8 wt%—Re, 30.9 wt%—α-Al_2_O_3_, 32.4 wt% η-Al_2_O_3_, 21.2 wt%—monoclinic ZrO_2_, 12.2 wt%—tetragonal—ZrO_2_) are completely different. η-Al_2_O_3_ content is 16.3% lower than in sample no. 5. The material is characterized by significant wear (see Table 6 and Figure 9B). The depth of wear is similar to the thickness of the layer. The micro-cutting abrasive mechanism is visible in Figure 10. However, taking into account the large amount of solid particles visible on the surface of the coatings, resulting in a roughness Ra of 10 μm, it is necessary to indicate a three-body abrasion mechanism in the first stage of the wear test, in which the material abrasion increases (Table 6). The wear mechanism in the first stage of the test should change, thanks to the reduction in the amount of large, hard ReB_2_ particles on the coating surface and consequently in the debris (during the wear process). This can be achieved by extending the milling time of the material immediately after the coating material synthesis process.

Investigations showed small phase changes in the coating after 6 months of storage. The XRD diffraction pattern in Figure 12 shows a small peak (H_3_O)(ReO_4_) [04-012-4121], which “is identical to phase called HReO_4_ in [38] (Database Comments). After 15 months, an increase in the presence of the phase (H_3_O)(ReO_4_) [04-012-4121] was found, which confirmed the reaction with water from moisture (Figure 12 and Figure 13). The presence of H_3_BO_3_ was not confirmed by the XRD method. The proposed concept, involving a composite coating composed of a matrix forming a eutectic that melts at a lower temperature and encapsulates ReB_2_ grains during atmospheric plasma spraying (APS), does not fully prevent ReB_2_ from interacting with atmospheric moisture. Nevertheless, it contributes to delaying the decomposition of both ReB_2_ and residual metallic rhenium. After 15 months of storage in a plastic bag, the phase composition revealed the presence of (H_3_O)(ReO_4_) (chemically equivalent to HReO_4_), while ReB_2_ and residual rhenium remained detectable. Researchers in [19] reported that after 12 months of storing ReB_2_ powder (produced by the mechanochemical method) in air without a protective atmosphere, the phase composition revealed the presence of HReO_4_ and boric acid in addition to ReB_2_. Other researchers [38] noticed that the composition of ReB_2_ powder (obtained by mechanical milling), after 2 months stored in ambient conditions in the presence of humidity and oxygen, contained the phases boric acid, ReO_3_, and HReO_4_. After 26 months, the powder completely decomposed [39].

The porosity of the coating has a decisive influence on the ReB_2_ reaction process. The ReB_2_ milling, greater dispersion of small particles, separation from pores, and greater densification of the coating material can result in improved chemical resistance of coatings. The issue is very similar to the presence of Al_4_C_3_ in steels and cast irons, where cutting off the compound from the porosity also improves the material properties [40].

## 5. Conclusions

The introduction of rhenium boride and rhenium as nucleation centers affected the crystallization process of the amorphous phase in the Al_2_O_3_ + ZrO_2_ + ReB_2_ + Re coating, as a result of which there is no amorphous phase in the APS coating.

The phase composition of APS coatings depends strongly on spraying conditions. The Al_2_O_3_ + ZrO_2_ + ReB_2_ + Re coating, with a high content of η-Al_2_O_3_ (about 50 wt%), is characterized by very high abrasion resistance.

ReB_2_ does not melt, which affects the higher roughness of the coating, and its initial particle size should be below 15 μm. The lower grain size of ReB_2_ particles in the Al_2_O_3_ + ReB_2_ phase could eliminate ReB_2_ clusters and reduce the porosity of APS coatings.

Chemical stability studies of the encapsulated ReB_2_ in Al_2_O_3_ indicate the presence of a small amount of reaction with water (from moisture) products. Reducing the number of pores in the coating can limit or even eliminate the ReB_2_ reaction with water.

## Figures and Tables

**Table 1:** Characteristics of the ReB_2_ + Al_2_O_3_ powder after crushing and grinding.

Properties	Al_2_O_3_ + ReB_2_ Powder—After Crushing and Milling
Density, g/cm^3^	5.29
Specific Surface, m^2^/g	0.6
Grain Size, μm	d_10_ = 0.3; d_50_ = 3.3; d_90_ = 15.1
Value Range, μm	0–112
Phases Composition	α-Al_2_O_3_—75.8% ReB_2_—23.2% Re_7_B_3_—1%

**Table 2:** Sieve analysis of the obtained agglomerates for 80 wt% Al_2_O_3_-ZrO_2_, 20 wt% Al_2_O_3_ + ReB_2_.

Agglomerate for Spray Coatings	˂45 μm	45–106 μm	106–212 μm	>212 μm
Fraction, %wt for 10 wt% Binder	36.4	32.9	30.3	0.4

Agglomerates for spray coating density is 4.61 g/cm^3^, bulk density—1.42 g/cm^3^, tapped density—2 g/cm^3^, flowability 16.7 s.

**Table 3:** Parameters for APS—NiAl coatings.

APS Coating	Current [A]	H_2_ Flow [L/min]	Ar Flow [L/min]	Spray Distance [mm]	Powder Container Rotation Speed [rpm]	Number of Cycles
NiAl	530	9	4.2	170	7.0	2

**Table 4:** Gase flows during the APS processes for 80 wt% Al_2_O_3_-ZrO_2_, 20 wt% Al_2_O_3_ + ReB_2_ coatings.

	Parameters of APS	Ar/H_2_ Rate	H_2_ Flow, [L/min]	Ar Flow, [L/min]
No. of Sample				
1		6.86	7	48
2		2.93	14	41
3 (500A)		2.93	14	41
4		10.8	5	54
5		12.8	5	64
6		14.8	5	74

Phase compositions of coatings are presented in Table 5.

**Table 5:** Results of the phase composition measurements of coatings using the XRD method (quantitative measurement—Rietveld method, wt%).

	Phase, wt%	ReB_2_	Re	α-Al_2_O_3_	η-Al_2_O_3_	ZrO_2_ Monoclinic	ZrO_2_ Tetragonal
No. of Sample							
1		1.2	1.7	10.7	55.8	14.4	16.2
2		0.6	2.5	4.6	66.9	9.9	15.5
3		0.7	3.6	3.9	71.0	7.6	13.2
4		1.2	1.5	14.2	48.7	19.0	15.5
5		2.6	0.8	30.9	32.4	21.2	12.2
6		2.6	0.6	28.0	30.6	22.9	15.3

**Table 6:** Average coefficients of friction and samples mass loss for the Al_2_O_3_ + ZrO_2_ + ReB_2_ + Re coatings and the 316L steel.

Material	Average Coefficient Friction	Sample Mass Loss, g	Counter-Sample Mass Loss, g
Al_2_O_3_ + ZrO_2_ + ReB_2_ + Re Sample no. 4	0.71 ± 0.05 *	0.0022	0.0003
Al_2_O_3_ + ZrO_2_ + ReB_2_ + Re Sample no. 5	0.79 ± 0.03 *	0.0145	0.0007
316L Steel	0.56 ± 0.02	0.0283	0.0013

* standard deviation.

## Data Availability

The original contributions presented in this study are included in the article. Further inquiries can be directed to the corresponding author.

## Abbreviations

The following abbreviations are used in this manuscript:

APS	Atmospheric plasma spray
CPCD	Confined-Plume Chemical Deposition
PLD	Pulsed laser deposition

## References

[1] Prashar G., Vasudev H., Thakur L., Thakur L., Vasudev H. (2022). Thermal spraying fundamentals. Process applications, challenges, and future market. Thermal Spray Coatings.

[2] Sathish M., Radhika N., Saleh B. (2024). Microstructure and dry sliding wear evaluation of functionally graded coating deposited via atmospheric plasma spray. Sci. Rep..

[3] Li L., Xie F., Wu X., He J., Li S. (2012). Microstructure and phase formation of atmospheric plasma sprayed YAG coatings. Surf. Coat. Technol..

[4] Heberlein J., Fauchais P., Boulos M. (2014). Thermal Spray Fundamentals: From Powder to Part.

[5] Dwivedi G., Viswanathan V., Sampath S., Shyam A., Lara-Curzio E. (2014). Fracture toughness of plasma-sprayed thermal barrier ceramics: Influence of processing, microstructure, and thermal aging. J. Am. Ceram. Soc..

[6] Shan X., Huang T., Luo L., Lu J., Cai H., Zhao J., Sheng G., Zhao X. (2023). Automatic Recognition of Microstructures of Air-Plasma-Sprayed Thermal Barrier Coatings Using a Deep Convolutional Neural Network. Coatings.

[7] Kim H.-J., Kim Y.J. (1999). Amorphous phase formation of the pseudo-binary Al_2_O_3_–ZrO_2_ alloy during plasma spray processing. J. Mater. Sci..

[8] Sodeoka S., Suzuki M., Inoue T. (2006). Thermal stability and mechanical properties of plasma sprayed Al_2_O_3_/ZrO_2_ nano-composite coating. Key Eng. Mater..

[9] Tarasi F., Medraj M., Dolatabadi A., Oberste-Berghaus J., Moreau C. (2011). Amorphous and crystalline phase formation during suspension plasma spraying of the alumina–zirconia composite. J. Eur. Ceram. Soc..

[10] Liu S., Zhu Y., Lai X., Zheng X., Jia R., Yuan X. (2019). Influence of Different Heat Treatment Temperatures on the Microstructure, Corrosion, and Mechanical Properties Behavior of Fe-Based Amorphous/Nanocrystalline Coatings. Coatings.

[11] Chen Y.-D., Yang Y., Chu Z., Chen X., Wang L., Liu Z., Dong Y., Yan D., Zhang J., Kang Z. (2018). Microstructure and properties of Al_2_O_3_-ZrO_2_ composite coatings prepared by air plasma spraying. Appl. Surf. Sci..

[12] Chraska T., Neufuss K., Dubský J., Ctibor P., Rohan P. (2008). Fabrication of bulk nanocrystalline alumina–zirconia materials. Ceram. Int..

[13] La Placa S.J., Post B. (1962). The crystal structure of rhenium diboride. Acta Crystallogr..

[14] Chung H.Y., Weinberger M.B., Levine J.B., Cumberland R.W., Kavner A., Yang J.M., Tolbert S.H., Kaner R.B. (2007). Synthesis of ultra-incompressible superhard rhenium diboride at ambient pressure. Science.

[15] Otani S., Korsukova M.M., Aizawa T. (2009). High-temperature hardness of ReB_2_ single crystals. J. Alloys Compd..

[16] Long R., Dai Y., Jin H., Huang B. (2008). Structural, elastic, and electronic properties of ReB_2_: A first -principles calculation. Phys. Res. Int..

[17] Portnoi K.I., Romashov V.M. (1968). State diagram of the rhenium-boron system. Porosh. Met..

[18] Maździarz M., Mościcki T. (2016). Structural, mechanical, optical, thermodynamical and phonon properties of stable ReB_2_ polymorphs from density functional calculations. J. Alloys Compd..

[19] Orlovskaya N., Xie Z., Klimov M., Heinrich H., Restrepo D., Blair R., Suryanarayana C. (2011). Mechanochemical synthesis of ReB_2_ powder. J. Mater. Res..

[20] Bliem P., Mráz S., Sen S., Hunold O., Schneider J.M. (2018). Self-passivating (Re,Al)B_2_ coatings synthesized by magnetron sputtering. Sci. Rep..

[21] Wicher B., Chodun R., Trzciński M., Lachowski A., Nowakowska-Langier K., Ibrahim S.H., Jaroszewicz J., Kubiś M., Grzanka E., Zdunek K. (2022). Application of the plasma Surface sintering conditions in the synthesis of ReBx-Ti targets for hard films deposition in magnetron sputtering technique. Int. J. Refract. Met. Hard Mater..

[22] Ivanov B.L., Wellons M.S., Lukehart C.M. (2009). Confined-Plume Chemical Deposition: Rapid Synthesis of Crystalline Coatings of Known Hard or Superhard Materials on Inorganic or Organic Supports by Resonant IR Decomposition of Molecular Precursors. J. Am. Chem. Soc..

[23] Rylski A. (2013). ReB_2_ coating on carbide and high speed steel substrates. Inżynieria Mater. (Mater. Eng.).

[24] Latini A., Barinov S.M., Rau J.V., Ferro D., Teghil R., Albertini V.R., Barinov S.M. (2008). Superhard Rhenium Diboride Films: Preparation and Characterization. Chem. Mater..

[25] Chrzanowska J., Hoffman J., Denis P., Giżyński M., Mościcki T. (2015). The effect of process parameters on rhenium diboride films deposited by PLD. Surf. Coat. Technol..

[26] Shen L., Tesfaye F., Li X., Lindberg D., Taskinen P. (2021). Review of rhenium extraction and recycling technologies from primary and secondary resources. Min. Eng..

[27] Nishiyama K., Nakamur T., Utsumi S., Sakai H., Abe M. (2009). Preparation of ultrafine boride powders by metallothermic reduction method 16th International Symposium on Boron, Borides and Related Materials. J. Phys. Conf. Ser..

[28] Czechowska K., Wrona A., Onderka B., Krzywiecki M., Warski T., Pęcak K., Czerny M., Jaworska L. (2023). Synthesis of composite powders and chemical resistance of the products to moisture. Int. J. Refract. Met. Hard Mater..

[29] Chitu T.M., Oulahna D., Hemati M. (2011). Wet granulation in laboratory scale high shear mixers: Effect of binder properties. Powder Technol..

[30] Baronskiy M.G., Tsybulya S.V., Kostyukov A.I., Zhuzhgov A.V., Snytnikov V.N. (2022). Structural properties investigation of different alumina polymorphs (η-, γ-, χ-, θ-, α-Al_2_O_3_) using Cr3+ as a luminescent probe. J. Lumin..

[31] (2000). Flat Products Made of Steels for Pressure purposes—Part 7: Stainless Steels.

[32] Sun J., Wang J., Zhou X., Dong S., Deng L., Jiang J., Cao X. (2018). Microstructure and thermal cycling behavior of plasma-sprayed LaMgAl11O19 coatings. Ceram. Int..

[33] Myerson A.S., Erdemir D., Lee A.Y. (2019). Crystal nucleation. Handbook of Industrial Crystallization.

[34] Johansen A., Schæfer T. (2001). Effects of interactions between powder particle size and binder viscosity on agglomerate growth mechanisms in a high shear mixer. Eur. J. Pharm. Sci..

[35] Nettleship I., Stevens R. (1987). Tetragonal zirconia polycrystal (TZP)—A review. Int. J. High Technol. Ceram..

[36] Golla B.R., Mukhopadhyay A., Basu B., Thimmappa S.K. (2020). Review on ultra-high temperature boride ceramics. Prog. Mater. Sci..

[37] Pantelis D.I., Psyllaki P., Alexopoulos N. (2000). Tribological behaviour of plasma-sprayed Al_2_O_3_ coatings under se-vere wear conditions. Wear.

[38] Wltschek G., Svoboda I., Fuess H. (1993). The Crystal Structure of Solid Perrhenic Acid Monohydrate. Z. Anorg. Allg. Chem..

[39] Granados -Fitch M.G., Quintana-Melgoza J.M., Juarez-Arellano E.A., Avalos-Borja M. (2018). Chemical stability of superhard rhenium diboride at oxygen and moisture ambient environmental conditions prepared by mechanical milling. J. Am. Ceram. Soc..

[40] Gilewski R., Kopyciński D., Guzik E., Szczęsny A. (2021). Shaping the microstructure of high-aluminum cast iron in terms of the phenomenon of spontaneous decomposition generated by the presence of aluminum carbide. Materials.

